# Complete Genome Sequence of a Colistin Resistance Gene (*mcr-1*)-Bearing Isolate of *Escherichia coli* from the United States

**DOI:** 10.1128/genomeA.01283-16

**Published:** 2016-11-10

**Authors:** Richard J. Meinersmann, Scott R. Ladely, James L. Bono, Jodie R. Plumblee, M. Carolina Hall, Linda L. Genzlinger, Kimberly L. Cook

**Affiliations:** a USDA Agricultural Research Service, Athens, Georgia, USA; b USDA Food Safety and Inspection Service, Athens, Georgia, USA; c USDA Agricultural Research Service, Clay Center, Nebraska, USA

## Abstract

Transmissible colistin resistance conferred by the *mcr-1* gene-bearing IncI2 plasmid has been recently reported in *Escherichia coli* in the United States. We report here the completed genome sequence of a second *E. coli* strain isolated from swine in the United States that carried the *mcr-1* gene on an IncI2-type plasmid.

## GENOME ANNOUNCEMENT

Liu et al. (1) recently described a gene, *mcr-1*, coding for a phosphoethanolamine transferase enzyme, which catalyzes a change in the colistin target to confer resistance, and is carried on an IncI2 plasmid in a strain of *Escherichia coli* with multiple antimicrobial resistances. The *mcr-1* gene has been recently found in the United States in human (2) and swine (3) isolates. The sequence for the *mcr-1* gene has been identical in every isolate yielding no phylogenetic signal. Therefore, the entire genomic sequence may be helpful for tracking the lineages carrying the gene.

As part of the National Antimicrobial Resistance Monitoring System (NARMS), a sample from Illinois of swine cecal contents was received and incubated overnight at 37°C in buffered peptone water (Acumedia; Neogen Corporation) with 2 µg/ml colistin (Sigma-Aldrich). This was followed by screening for the *mcr-1* gene using a PCR as described by Liu et al. (1). A PCR amplification product of expected size was noted and the culture plated onto MacConkey agar (Acumedia) supplemented with 2 µg/ml colistin for isolation. Presumptive positive colonies were rescreened by PCR and were identified to the species level using the Vitek 2 system (bioMérieux).

A colistin-resistant isolate identified as *Escherichia coli* was found to be PCR positive for the *mcr-1* gene. DNA was prepared from the isolate, and the genomic sequence of the strain was determined by sequencing with Pacific Biosciences P6-C4 chemistries and assembled with HGAP3 (4) into a circular chromosomal contig of 4,668,621 bases and one circular plasmid contig of 63,329 (pSLy21) bases. The genome was also sequenced by Illumina MiSeq using the Nextera XT library prep kit and the MiSeq 2000 500-cycle paired-end (PE) kit. The PacBio sequences were used as a reference for mapping the Illumina data, and the contig was edited as needed. The mean Illumina data coverage for the chromosome was 98-fold and was 181-fold for the plasmid.

The chromosome carried genes for multilocus sequence type (MLST) ST-132 (5). The O serotype could not be determined from the sequence, the H type gene was H32, and the virulence factors *gad* and *astA* were found (6) (http://www.genomicepidemiology.org). pSLy21 carried an IncI2 replication initiation protein gene and carried the *mcr-1* gene that was 100% identical to all the *mcr-1* genes found in GenBank. No other antimicrobial resistance genes were found either on the plasmid or chromosome.

### Accession number(s).

Sequences were deposited in GenBank under the accession numbers CP016404 (chromosome) and CP016405 (pSLy21).
